# Enduring impact of conflict on mental health and gender-based violence perpetration in Bougainville, Papua New Guinea: A cross-sectional study

**DOI:** 10.1371/journal.pone.0186062

**Published:** 2017-10-25

**Authors:** Rachel Jewkes, Nwabisa Jama-Shai, Yandisa Sikweyiya

**Affiliations:** 1 Gender & Health Research Unit, Medical Research Council, Pretoria, South Africa; 2 School of Public Health, University of the Witwatersrand, Johannesburg, South Africa; Public Library of Science, FRANCE

## Abstract

**Background:**

Violence against women is often exacerbated by war, but most civilian research has investigated short term impact. We describe the conflict experiences of men and women from the general population of Bougainville Papua New Guinea, perceptions of the enduring impact of conflict, and the associations between these and the major health and development problems on the islands: mental ill-health and violence against women.

**Methods:**

Fourteen years after the end of the decade long civil war, we conducted a household survey with a random sample of adult (n = 864) men and (n = 879) women living in Bougainville. The interviews were mostly conducted face-to-face, with very sensitive questions self-completed.

**Results:**

Mental ill-health was highly prevalent, 37.8% of women and 32% of men had high levels of depressive symptomatology, 34.4% of men abused alcohol and 15.1% of women and 24.6% of men had high levels of PTSD symptoms. Among women, 23.3% had been raped in the year prior to the interview and 33.3% had experience physical or sexual partner violence. The prevalence of exposure to trauma during the civil war was very high and many of the men and women experienced lingering impact of conflict. Multiple logistic regression models showed that war trauma was associated with PTSD symptoms in women and PTSD symptoms, alcohol abuse and depressive symptoms in men. The perceived enduring impact of conflict was associated with depressive symptoms in men and women, problem drinking and suicidal thoughts in women and drug use in men. The perceived enduring conflict impact was associated with perpetration of past year rape and physical and/or sexual partner violence.

**Discussion:**

The Bougainville civil war had a devastating impact on the population’s lives. Reversing this legacy is essential but requires addressing what is perceived as the enduring social, economic and psychological impact of the conflict and a major focus on prevention of violence against women.

## Introduction

The impact of conflict on violence against women and girls has been a growing area of concern over recent years. There has been a substantial focus on rape in conflict, which was drawn into sharp focus by the 2014 Preventing Sexual Violence Initiative Summit in London. Evidence points to rape being a marked feature of conflict in some countries, but not in all conflicts[1]. There also is a growing body of evidence to indicate that intimate partner violence (IPV) and violence against children are increased during and in the short term post-conflict[2–6]. IPV is exacerbated by the conflict in multiple ways, but there is evidence from multiple countries to show that men and women’s experience of trauma in war is associated with a greater propensity to perpetrate IPV as well as a heightened risk of victimisation [2, 7].

The impact of war on combatants including military veterans has been comparatively well studied. It is well established that involvement in war can result in post-traumatic stress disorder and depression and that these are associated with a greater risk of men perpetrating violence against an intimate partner [8, 9]. Studies of war impact on the general population (which may include some ex-combatants) post-conflict in countries such as Uganda and Liberia, and lands with current conflict such as the occupied Palestinian territories, have documented exposure to a range of terrifying experiences and have shown that these have enduring impact [2, 6, 7]. They are associated with PTSD and depression, as well as violence perpetration. Researchers have also identified a range of other impacts of war which influence IPV including deepening poverty, disrupted families, reduced functioning of police and justice systems, growing acceptability of use of violence, and undermining of traditional masculinities [10–12]. In the case of the latter, war often reduces men’s ability to provide for their family and their or their family’s victimisation undermines their self-perception of being strong and in control, and in response violence may be used in an attempt to grasp back the lost control and respect [10, 12].

There are some notable gaps in research around violence against women and impact of war. There has been comparatively little research among the general population of countries on violence against women in the medium and long term post-conflict. If violence is increased during conflict, it is not known how long this persists for nor whether it declines over time without intervention. Further the social impact of war and its impact on violence against women has been thinly described. It has been expressed in terms of poverty or distruption of social and political systems, yet there is a need for a more graded understanding of this impact and this has direct implications for post-war reconstruction and violence prevention. Impact of war is largely investigated in research in terms of war trauma, which is often read through a lens of mental health impact, but there is a need to understand if there is the impact of post-war trauma and loss in violence against women more generally.

Drawing on data from a general population survey conducted in Bougainville, Papua New Guinea 14 years after the formal cessation of hostilities, this paper extends understanding of the enduring impact of conflict on mental health and perpetration of violence against women. We investigate the hypothesis that the impact of conflict on social lives of men and women in the general population endure long after the end of a conflict and that this enduring impact is associated with poorer mental health and enhanced risk of violence perpetration, and that these are not just explained by symptoms of post-traumatic stress.

### Bougainville and the conflict

In 2012, Bougainville province of Papua New Guinea was home to about 175 000 people from 19 ethic groups who lived on two main islands, Buka and Bougainville Island, and 168 smaller islands (atolls). The islands had thick mangrove swamps around the coast and forest inland of hard wood with banana and coconut palms, broken by rivers flowing down the slopes of three volcanos. There were very few roads, bridges, clinics or schools on Bougainville and many people lived in villages accessed by walks of many hours through thick forest or by boat. The economy was largely based on subsistence agriculture, although the island has the world’s largest copper mine.

From 1988 for a decade Bougainville had a devastating civil war. In the first two years separatist forces of the Bougainville Revolutionary Army (BRA) fought the Papua New Guinea army and police. Then in May 1990 the Papua New Guinea government pulled out the army as well as all government services and resources. Schools and hospitals, banks and businesses all were closed and for four years Papua New Guinea imposed an air and sea blockade, including blocking medical supplies [13]. For a decade the health and education systems effectively ceased to function and there was vicious fighting between the BRA and various armed factions [13]. The general population was massively affected by the loss of health and other services as well as the wide spread rape, killings, beatings, burning of villages and acts of mutilation (including forced circumcision). Families were torn apart, non-Bougainvillians forced off the islands, and the more educated and wealthier Bougainvillians were targeted and harassed, imprisoned, tortured or murdered[14]. An estimated 10,000–15,000 people were killed, and 64,000 detained in ‘care centres’ where many human rights abuses occurred [15].

The war was formally ended in 1998, but peace building was incremental and in 2005 resulted in establishing the Autonomous Bougainville Government. This research was conducted in 2012, which was 14 years after the official peace but in some parts of Bougainville this was still fragile and disarmament incomplete. Post-conflict reconciliation processes facilitated some of the reintegration of the “lost” generation of young men who fuelled the war, and there have been claims that rape and violence against women have fallen as a result [16], although the evidence base for this is unclear. Gender-based violence is fundamentally fuelled by gender identity and cultural norms around the use of violence [17]. Not withstanding matrilineal traditions in the Pacific, gender inequity is pronounced and many men in Melanesia see violence as the normal and desirable masculine way of resolving conflict or expressing anger [18–21]. Men are often socialised from early childhood to interact forcefully with others [22, 23]. The belief that violence is an entirely appropriate corrective measure for wives is widespread, and male sexual entitlement, including rape of women, is often culturally scripted [24].

At the time of the survey in 2012 Papua New Guinea’s Criminal Code did not have an offence of domestic violence (it adopted this in 2013), but rape was proscribed under the Sexual Offences Amendment Act of 2003. The very high degree of State tolerance of violence against women was visible in the marriage law (Matrimonial Causes Act of 1963) which only permitted divorce if ‘during a period of not less than one year, {the other party had} habitually been guilty of cruelty’, had committed rape, sodomy or bestiality’ and ‘within a period of one year immediately preceding the date of the petition…had been convicted of i) having attempted to murder or unlawfully to kill the petitioner or ii) having committed an offence involving the intentional infliction of grievous bodily harm on the petitioner or the intent to inflict grievous bodily harm on the petitioner’.

The survey aimed to advance understanding on the prevalence, risk factors and health consequences of violence, particularly gender-based violence, in Bougainville, Papua New Guinea. It was conducted in 2012 as part of the UN Multi-country Cross-sectional Study on Men and Violence, which was developed by Partners for Prevention, a UNDP, UNFPA, UN Women and UNV regional joint programme for gender-based violence prevention in Asia and the Pacific, and was an international collaboration with the Medical Research Council of South Africa and the research teams in each country. In this paper we describe the historical impact of conflict on the general population of re-integrated combatants and non-combatants of Bougainville; the enduring impact of conflict on the lives of people in the general population, including on their mental health; and investigate the perceived enduring impact of conflict on gender-based violence perpetration, independent of that mediated by PTSD.

## Methods

A two stage proportionate stratified design was used to identify a representative sample. The Papua New Guinea 2010 national census was used as the primary sampling frame. The census data provided by the Papua New Guinea National Statistics Office gave an up to date description of all census units, household count and a map of household location. Sample selection used probability proportionate to size method, with the sample stratified by census unit size and by district. The following procedure was used in sample selection. The census units were divided into four categories based on size (number of households) and divided into three districts of Bougainville (north, central and south). The size categories were <20 households, 20–39, 40–79 and 80 and over. Census units with less than 20 households and those on atolls (very inaccessible islands) were dropped from the sampling frame, this amounted to 9.6% of the potential Bougainville population. After a random start, census units were selected using probability proportionate to size, within each stratification category. The number of census units selected in each category was proportionate to the size (number of households) of the category. After selection, the census units were examined and three that were very inaccessible geographically (requiring a walk of an estimated 5 or more hours) or politically (due to instability) were replaced using the same selection procedure. The sample selection was undertaken by the National Statistics Office of Papua New Guinea.

The initial plan for the study was to interview 1500 men and 1500 women in Bougainville, conducting the research in 150 census units with 20 households approached for interview in each census unit. However this plan was adjusted to take into account the realities on the ground and an expectation of a high rate of being unable to complete interviews in selected households. The assumptions of the sample size calculation were that we would assume that the prevalence of perpetration or victimisation of physical or sexual intimate partner violence was 35%, with alpha = 0.05 and power of 0.80, the sample size required for a precision of = /- 5% was 726 per gender.

Eligible interviewees were men and women aged 18–49 years, normally resident in the sampled household and apparently mentally competent to complete the questionnaire. If there were more than one potentially eligible respondent in a household one would be randomly selected. For a census unit to be eligible for inclusion in the sample it should have >19 households, be on Bougainville or Buka islands or on a large neighbouring island (i.e. not be an atoll) and should be accessible with less than 5 hours of walking. During the process of examining the census unit size, it was realised that there were very few census units with 100 households on the island and a substantial proportion had under 50 households. Thus, the sample was stratified by census unit size; we were not able to implement the plan of interviewing in 20 households in the smallest census unit category. In this category we interviewed in 10 households and for the presentation of national prevalence estimates we applied a sampling weight to the data.

At the point of interview, a household that did not have an eligible interviewee was not replaced. If the sampled household member was not at home at the first visit, two further attempts were made to interview the sampled interviewee. No substitutions were made if the selected interviewee could not be interviewed.

In total 1014 households were selected for conducting a male interview and determined to have an eligible man residing in them. Among these there were 864 (85.2%) that an interview could be completed within. For female interviews, 1034 households were identified with an eligible female household member and in 879 (85.0%) an interview was conducted. The main reason for non-response was being unable to make contact with the household or selected person to do the interview, refusal was very uncommon, with only about 1 in 5 of those not interviewed having actually refused an interview, or started but declined to complete one.

### Questionnaire

The core questionnaire which was developed for the UN Multi-country Study on Men and Violence with inputs from the Regional Research Advisory Group was used by all participating countries, however, each participating country added certain questions. For the Bougainville survey, there were two questionnaires, one for men and another for women, which were very similar.

The face validity and appropriateness of the core questionnaire for the cultural context was initially tested at a meeting of the Papua New Guinea National Working Group where it was examined question by question. Then in a process where four experienced local interviewers spent two days going through question by question discussing the face validity of items. They suggested new items needed to enhance the local relevance of the questionnaire. The interviewers each undertook a test interview with the Tok Pisin translation of the questionnaire and then corrected the translation through a process of group discussion to ensure it expressed the ideas of the English questionnaire appropriately in the Bougainville Pisin. These questionnaires were then retested and checked by members of UN staff and the National Working Group before being finalised.

The questionnaires included questions on the social and demographic background of participants. Full details of the questions are presented elsewhere [25, 26]. Questions on emotional, financial and physical intimate partner violence perpetration (men) and victimisation (women) were taken from the instrument developed in the World Health Organisation’s Multi-Country Study [27]. Rape perpetration (men) and victimisation (women) was assessed. The men’s questions were a refinement on the ones used in South Africa [28]. Physical IPV was measured for both men and women with five behaviourally specific items that asked about actions of a current or former partner in the last year: was slapped or had something thrown at her that could hurt her; pushed or shoved; hit with a fist or something else that could hurt her; kicked, dragged or beaten up; current/former partner threatened to use or actually used a gun, knife or other weapon against her; these were developed from Garcia-Moreno et al 2005; each had never, once, few, many response options. For men the questions were inverted so they asked about perpetration and not violence experience. Sexual IPV was measured with two items also asking about the past 12 months: Has a current or previous husband or boyfriend ever physically forced you to have sex when you did not want to? Have you ever had sex with a current or previous husband or boyfriend when you did not want to because you were afraid of what he might do? For men, the first question was the same, but the second one slightly different in that it asked ‘Have you ever had sex with your current or previous wife or girlfriend when you knew she didn’t want it but you believed she should agree because she was your wife/partner?’. The composite measure was ‘yes’ if any of these had occurred.

Non-partner rape perpetration was measured in men with two items that asked about having forced a woman who was not your wife or girlfriend at the time to have sex; having had sex with a woman who was too drunk or drugged to indicate whether she wanted it. Two futher items asked the same but with the formulation 'with other men'. Victimisation was measured in women with 3 items asking about being forced or persuaded to have sex against your will by a man who wasn’t your husband or boyfriend; forced to have sex with a man who was not a husband or boyfriend when too drunk or drugged to refuse; forced or persuaded to have sex against your will with more than one man at the same time.

Ten items from the short form of the CES-D scale were used to assess depressive symptomatology [29]. The category ‘highly symptomatic’ was based on a cut point score of 0–9 versus 10 or more. We asked about suicidal thoughts in the last month. We asked about alcohol using questions adapted from the AUDIT scale [30]. These captured frequency of drinking alcohol, numbers of drinks typically consumed, binge drinking, failing to do what was normally expected due to drinking and guilt/remorse after drinking. We asked “How many times have you used drugs in the last 12 months?” as a single item on drug use. We used a 15 items Harvard Trauma Questionnaire to measure symptoms of Post-Traumatic Stress Disorder (PTSD). Each item responses were scored from 1–4, and the total across the 15 items was summed and then divided by 15 to get an overall PTSD symptom score. We determined a person to be ‘highly symptomatic’ if this score was 2.5+.[31]

We had questions on exposure to violence as a victim during the Bougainville conflict. These were developed specifically for this study in conversation with research staff and the national working group. For men, we also asked about perpetrating violence during the conflict. These were not asked for women as very few women were combatants. There were two domains to the war trauma questions, one was four items on violence witnessed in conflict and one was 13 items on traumatic experiences. On advice from the working group and interviewers, only women were asked about being forced to have sex with a family member or friend and only men about forced circumcision so each gender was asked 12 questions. The two domains were scored and then added together to give an overall war trauma score and this was categories for the analysis into no trauma, one type and more than one experience.

We developed questions on the enduring social consequences of the conflict after reading descriptions of these and discussing them with the main interviewer group and Working Group. The perceived enduring impact of conflict measure was derived by scoring not being able to return to school after the conflict, unable to keep employment and eight other perceived conflict impact items (shown in table below and S1 File). Each item contributed 0 or 1 to the score giving a maximum score of 10. It was divided into three categories: low impact was a score of 0 or 1, medium impact scored 2–4 and high impact 5 and over. Cronbach’s alpha 0.70 for men and 0.916 for women.

The fieldworker training and pilot testing of the study were conducted in July-August 2012 over two weeks (11 working days). Eight working days were spent in the class room and three days were spent in the field for the pilot study with interviewers deployed to conduct interviews in villages not selected for the main study. Each interviewer was required to complete four interviews during this time. Interviewers did not come from selected villages (or interview where they came from) and were sex matched to interviewees.

### Ethical considerations

The study received ethics approval from the South African Medical Research Council Ethics Committee. Permission to conduct the study in Bougainville was also granted by the Chief Administrator on behalf of the Autonomous Bougainville Government. Participants (men and women) were invited to participate and were told they were free to decline and that there would be no repercussions from doing so, and that they may withdraw at any stage, skip any question and that there will be no direct benefits for them from participation. All participants who volunteered to participate gave written informed consent.

The UNDP and National Statistics Office staff conducted community mobilisation in the selected census units, first presenting the study to Chiefs in all the selected villages. In keeping with the WHO (2001) guidelines on “Ethical and Safety Recommendations for Domestic Violence Research”, the study was presented to the communities and prospective participants as a ‘Family, Health and Safety Study’ and not as a survey on gender-based violence.

Anonymity was imperative because of the sensitive nature of some of the questions. Anonymity of the participants’ responses was ensured by the use of Personal Data Assistants (PDAs) to collect data from participants. Most of the questionnaire was interviewer administered (all for women), but the section on violence perpetration and conflict was self-administered with responses directly entered on the PDA. This enhanced confidentiality. Although literacy levels were low, we tested PDA use with men in villages outside Buka and found it acceptable. Fieldworkers were present to assist in all self-completed sections if needed and did not report problems. Further information on ethical issues can be found elsewhere [32].

### Data analysis

The study design provided a sample that required weighting because of the 50% sample in small villages, for this reason Ns are not provided for cells in the tables. All procedures used Stata 13 and took into account the structure of the dataset, with stratification by district and and the enumeration areas (EAs) as clusters. Variables were summarized as percentages (or means), with 95% confidence limits calculated using standard methods for estimating confidence intervals from complex multistage sample surveys (Taylor linearization).

In order to use the IPV variables the multivariable regression analyses were performed on data from 746 men and 793 women who had ever been married, partnered or had a girlfriend or boyfriend. In order to investigate the relationship between the perceived enduring impact of conflict and mental health and violence perpetration, we first calculated the prevalence of the independent variable per category of the outcome variable for a set of putatively causal mental ill-health and violence perpetration outcomes, by gender. Then we used random effects logistic regression models to model each outcome variable, by gender. The independent variables were the three level war trauma variable, the three level perceived conflict impact measure, ever having been raped by a non-partner (asked of men and women) and for women, having ever experienced more than one episode of physical or sexual IPV. All models were adjusted for age and district.

The model for alcohol abuse was adjusted for depressive symptoms. The model for depressive symptoms was presented in two versions, without and with adjustment for PTSD symptoms and problem alcohol drinking. A process of elimination of non-significant variables was not used in these models.

The model for PTSD symptoms in men was unable to run model with categorical variable for war trauma experience due to its exceptionally high prevalence, we instead used the two scored variables (continuous) from which the categorical variable was derived. The Cronbach’s alpha for the witnessing abuse variables was 0.74 and for the personal experiences in conflict was 0.79. The model for suicidal thoughts and for past year drug use are only presented for one gender as the thoughts/behaviour was low prevalence in the other gender. Both models included a term for depressive symptoms and for both backwards elimination was used to ultimately retain variables at p = or <0.05 due to the lower prevalence of the outcome.

To determine the experience of perceived enduring impact of conflict on violence perpetration, the models included terms for age, district, depresssion, PTSD, drug use and alcohol problems, as well as the war trauma and conflict impact variables. Elimination of non-significant variables was not used.

## Results

The mean age of the men and women interviewed was 31 years (Table 1). Most of those interviewed had some schooling, but the majority (53.3% of women and 54.6% of men) had only attended primary school. Women were much less likely than men to be employed (48.1% v. 73.7%). Two thirds of research participants were married or cohabiting, but 26.9% of men and 16.7% of women had either never been partnered or were currently unpartnered (but previously married).

**Table 1:** Characteristics of respondents by gender.

	Female			Male		
	(n = 870)	95% confidence intervals		(n = 873)	95% confidence intervals	
*District of residence*						
North	34.3%	30.6%	38.1%	37.7%	34.1%	41.3%
Central	31.7%	27.2%	36.5%	28.2%	25.4%	31.3%
South	34.1%	29.9%	38.5%	34.1%	30.1%	38.4%
Mean age (95%CI)	31.4 (30.6, 32.2)			31.5 (30.8, 32.3)		
*Education*:						
None	6.3%	4.8%	8.3%	2.9%	1.8%	4.6%
Primary	53.3%	48.8%	57.6%	54.6%	50.3%	58.8%
Some secondary	12.3%	9.4%	15.9%	13.7%	11.0%	16.9%
Secondary complete	20.7%	17.1%	24.7%	18.4%	15.6%	21.5%
Any tertiary	7.4%	5.7%	9.6%	10.5%	7.9%	13.8%
Employed: in last year	48.1%	43.9%	52.4%	73.7%	68.6%	78.3%
employed but not in last year	44.0%	40.1%	48.0%	17.8%	14.8%	21.4%
Never employed	7.9%	5.9%	10.4%	8.4%	5.8%	12.1%
*Relationship status*						
Never partnered	9.4%	6.5%	13.3%	15.3%	12.3%	18.8%
Currently married / cohabiting	69.9%	65.3%	74.2%	63.6%	59.2%	67.9%
Currently has partner but not living together	13.4%	11.3%	15.8%	9.5%	7.6%	11.9%
Previously married/partnered	7.3%	5.7%	9.3%	11.6%	9.6%	13.9%
*Exposure to non-partner rape*						
Raped in the past 12 months (victimisation, women; perpetration, men)	23.3%	19.6%	27.5%	25.1%	21.1%	29.5%
Ever raped by a non-partner	15.8%	12.8%	19.4%			
Rape experience of men				6.7%	4.9%	9.0%
Exposure to more than one episode of physical or sexual IPV	57.9%	53.0%	62.6%			
Past year physical or sexual IPV (victimisation, women; perpetration, men)	33.3%	28.9%	37.9%	33.4%	29.1%	38.1%
High level of depressive symptoms	37.8%	32.5%	43.4%	32.0%	28.4%	35.9%
Suicidal thoughts in the last 4 weeks	8.2%	5.8%	11.5%	3.0%	1.9%	4.7%
Problem drinking	7.3%	5.1%	10.3%	34.4%	31.1%	37.9%
Drug use in last 12 months	2.6%	1.7%	3.9%	17.3%	14.0%	21.0%
Proportion exposed to trauma that might lead to PTSD	79.5%	75.4%	83.1%	78.6%	74.2%	82.5%
Proportion of total with high levels of PTSD symptoms	15.1%	12.2%	18.5%	24.6%	21.1%	28.4%

Many of the interviewees had previous exposure to violence. Rape was particularly common, with 23.3% of women and 25.1% of men having experienced or perpetrated rape in the 12 months prior to the interview. 6.7% of men disclosed having ever themselves experienced rape. Intimate partner violence was also very common and 33.3% of women has experienced physical or sexual partner violence in the previous year and 34.3% of men reported perpetrating this. In total 78.5% of men and 79.6% women had experienced trauma of a life threatening nature that would place them at risk of PTSD.

Mental ill-health was highly prevalent. Women disclosed more suicidal thoughts than men, with 8.2% of woman having had suicidal thoughts in the previous month compared to 3% of men. 37.8% of women and 32% of men had high levels of depressssive symptomatology. Men were much more likely to disclose substance abuse than women, with 34.4% of men having problem drinking and 17.3% having used drugs in the last year. In total 15.1% of women and 24.6% of men had high levels of PTSD symptoms.

The prevalence of exposure to traumatic war experiences is shown in Table 2. The prevalence of exposure was very high and for each experience, higher for men than women. Over a third of women and half of men had witnessed someone being killed in the conflict, and nearly half of all women interviewed, and two thirds of men had witnessed someone being seriously injured. The men and women had personally experienced a range of traumatic experiences, for example 8.3% of men had themselves been sexually violated, 40.4% had been forcibly circumcised and 16.2% had been beaten or tortured. Over a third of women and nearly half of men had themselves been interned in a ‘care centre’, and nearly half of the men who were interned were punished in the care centre for having a relative who was a combatant.

**Table 2:** Conflict experience by gender.

		Female			Male		
		(n = 870)	95% confidence intervals		(n = 873)	95% confidence intervals	
**Violenced witnessed during conflict**	Someone being beaten	46.8%	42.6%	51.1%	63.2%	58.3%	67.8%
	Someone killed	36.5%	32.2%	41.0%	50.8%	46.5%	55.2%
	Someone seriously injured	46.3%	41.3%	51.3%	65.1%	60.4%	69.5%
	Rape or sexual violation of men or women	13.6%	11.1%	16.7%	30.0%	26.7%	33.5%
**Personal experience of violence and abuse related to conflict**	Forced into marriage	5.7%	4.0%	8.1%	13.3%	10.9%	16.2%
	Beaten by the PNGDF or the Resistance or BRA	2.9%	1.9%	4.5%	16.3%	13.8%	19.1%
	Seriously injured	4.4%	3.0%	6.3%	20.2%	16.9%	23.9%
	Forced to have sex, raped or otherwise sexual violated	2.0%	1.1%	3.5%	8.3%	6.4%	10.7%
	Forced to have sex with a family member or friend	0.9%	0.4%	1.8%			
	Experience sexual abuse with foreign objects	0.4%	0.1%	1.8%	5.7%	4.2%	7.7%
	Detained or imprisoned	1.8%	1.0%	3.2%	17.2%	14.4%	20.4%
	Beaten or tortured	2.6%	1.6%	4.0%	16.2%	13.6%	19.3%
	Interned in a care centre	37.5%	31.6%	43.7%	46.9%	41.2%	52.6%
	Punished in a care centre because your male relative was in the bush	8.4%	6.5%	11.0%	22.4%	18.5%	26.7%
	Forced to separate from your parent/spouse	3.0%	1.9%	4.7%	14.1%	11.8%	16.9%
	Experience verbal or emotional abuse	13.6%	10.9%	17.0%	38.8%	35.2%	42.5%
	Forced circumcision				40.4%	35.2%	45.9%
**Men's involvement in conflict**	Involved in combat				29.2%	25.2%	33.6%
	Beat women or participated in beating women				12.2%	9.8%	15.1%
	Raped, or otherwise participated in rape of a woman				7.6%	5.7%	10.1%
	Forced, or participated in forcing, a woman into marriage				7.8%	6.0%	10.1%
	Killed a man or a woman				9.4%	7.4%	11.9%
**Contextualising the violence and abuse**	Experience any of these (the above) before the conflict	32.4%	28.4%	36.6%	57.0%	51.9%	61.9%
	Experience any of these (the above) after the conflict	23.4%	19.3%	28.1%	49.0%	44.7%	53.4%

Many of the men interviewed had been combatants (29.2% overall) and nearly one in 10 (9.4%) had killed in the course of this. Many had participated in acts of violence against women, with 20% of men having either beaten, raped or forced a woman into marriage during the conflict.

Overall 87.6% of men and 59.5% of women had experienced some form of trauma during the war. A high proportion of men and women (57.0% and 32.4%) disclosed that they had also experienced one of more of these forms of violence before the conflict started, and similarly many experienced them afterwards (49.0% of men and 23.4% of momen).

The conflict was perceived to have had an enduring impact on the lives of many of those interviewed (Table 3). Men’s education was reported as particularly affected, with 52.7% unable to complete their education due to the conflict. Women also perceived more impact on employment, with 29.8% of women and 20.5% of men perceiving themselves to be unable to keep employment due to impact of the conflict. In most other respects perceived conflict impact did not differ significantly between men and women. A third of those interviewed reported that there was a continuing lack of peace in their village due to the conflict and 20.7% of men and 27.6% of women disclosed continuing strife in their family. Between a quarter and a third reported enduring difficulties in their intimate relationships, controlling their aggression, with trust and in normal social relations in the community due to conflict. The prevalence of disability due to the conflict was also high, affecting more men (13.8%) than women (6.9%).

**Table 3:** Enduring consequences of conflict by gender.

	Female			Male		
	(n = 870)	95% confidence intervals		(n = 873)	95% confidence intervals	
Unable to complete education due to conflict	0.0%			52.7%	48.0%	57.5%
Unable to keep employment due to conflict	29.8%	24.9%	35.3%	20.5%	17.6%	23.7%
Continuing lack of peace in village or area	36.6%	31.5%	42.1%	38.9%	35.0%	42.9%
Continuing strife in family	27.6%	23.0%	32.7%	20.7%	17.8%	24.0%
Drinking or using drugs to forget the trauma of conflict	21.6%	17.1%	26.9%	24.3%	21.4%	27.4%
Difficulty having a good relationship with a (wo)man	26.6%	22.3%	31.5%	21.7%	18.7%	25.0%
Difficulty controlling aggression	32.7%	27.9%	37.9%	29.0%	24.9%	33.5%
Difficulty in normal social relations in the community	34.1%	29.2%	39.5%	33.8%	29.7%	38.3%
Unable to trust anyone	35.0%	29.8%	40.6%	36.6%	32.4%	41.1%
Disabled as a result of the conflict	6.9%	3.7%	12.5%	13.8%	11.0%	17.2%

The perceived enduring impact of conflict was scored and about a third of women were placed in each of the three (low, medium and high) impact categories. Most of the women reported lower enduring impact (56.5%) but nearly a third (28.3%) reported high impact. Among the men, only 35.8% reported low enduring impact of conflict and 25.7% reported high impact.

The associations between PTSD symptomatology and war trauma and violence against women are shown in Table 4. In women, PTSD symptomatology was associated with exposure to severe partner violence and non-partner rape, and trauma from war. In men, it was associated with war trauma. The logistic regression models of depressive symptomatology show that war trauma experiences were associated with this in men, directly and indirectly through their association with PSTD symptoms. The perceived enduring impact of conflict was associated with depressive symptoms in men and women, again both directly and through association with PTSD symptoms.

**Table 4:** Prevalence of conflict trauma, experience of rape and IPV among men and women with (and without) PTSD and associations between trauma exposure in conflict, physical and sexual violence and PTSD.

	PTSD symptoms in women						PTSD symptoms in men*						
	High symptoms	Low or none	aOR	95%CI		p value	High symptoms	Low or none	Variable used in model	aOR	95%CI		p value
Traumatic experiences in conflict: none	16.8%	44.2%	1.00				0	16%					
1	10.5%	12.3%	1.88	0.84	4.18	0.122	2.3%	12.7%	Witnessed abuse in conflict (score)	1.34	1.17	1.54	<0.001
2+	72.7%	43.5%	3.18	1.72	5.90	<0.001	97.7%	71.3%	Personal experiences of conflict trauma (score)	1.20	1.12	1.28	<0.001
>1 episode of physical or sexual IPV	84.8%	52.9%	2.16	6.84	5.61	<0.001							
Raped by a non-partner	31.8%	13.1%	1.41	3.98	3.37	0.001	9.7%	5.8%	Ever raped	1.22	0.63	2.36	0.550

* we are unable to run model with categorical variable for war trauma experience due to their exceptionally high prevalence, instead use the two scored variables (continuous) from which the categorical variable was derived. The %s shown are for the traumatic experiences in conflict categorical variable

The associations between problem drinking, war trauma and enduring conflict impact are shown in Table 5. War trauma experiences were associated with problem drinking in men and the perceived enduring impact of trauma was associated with problem drinking in both men and women. Both non-partner rape and severe intimate partner violence were associated with depressive symptoms and alcohol abuse in women (Tables 5 and 6). The table also shows factors associated with suicidal thoughts in women, and the perceived enduring conflict impact was associated, as well as depressive symptoms. In men, past year drug use was also associated with perceived enduring conflict impact, as were depressive symptoms and having been raped.

**Table 5:** Prevalence of conflict trauma, perceived enduring impact of conflict, rape and IPV and mental ill health among men and women with (and without) substance abuse and suicidal thoughts, and associations between these and substance abuse and suicidal thoughts.

	Alcohol abuse in women						Alcohol abuse in men					
	**Alcohol abuse**	**None**	**aOR**	**95%CI**		**p value**	**Alcohol abuse**	**None**	**aOR**	**95%CI**		**p value**
	**%**	**%**					**%**	**%**				
Traumatic experiences in conflict: none	39.5	40.5	1.00				5.7	15.9	1.00			
1	17.1	11.7	1.14	0.47	2.79	0.774	5.1	13.1	1.04	0.47	2.31	0.915
2+	43.4	47.8	0.60	0.27	1.33	0.211	89.2	71	3.53	1.91	6.51	<0.001
Enduring impact of conflict: none	34.2	57.7	1.00				26.1	38.5	1.00			
1	7.9	12.9	1.22	0.43	3.50	0.71	31.8	37.4	1.04	0.71	1.51	0.838
2+	57.9	29.3	3.15	1.48	6.70	0.003	42.0	24	1.70	1.11	2.60	0.015
>1 episode of physical or sexual IPV	75.7	56.5	2.27	1.08	4.79	0.031						
Raped by a non-partner	40.3	13.8	4.31	2.16	8.59	<0.001	9.5	5.3	1.19	0.66	2.14	0.562
PTSD symptoms	18.2	13.4	0.39	0.16	0.96	0.041	30.4	19.2	1.18	0.82	1.71	0.38
Depressive symptoms	61	36	2.41	1.26	4.63	0.008	39.5	27.9	1.43	1.04	1.98	0.029
	Suicidal thoughts in past month in women***						Drug use in past year in men**					
	**Suicidal thoughts**	**None**	**aOR**	**95%CI**		**p value**	**Drug use**	**None (%)**	**aOR**	**95%CI**		**p value**
	**%**	**%**					**%**	**%**				
Traumatic experiences in conflict: none	23.0	42.0					8.3	13.2				
1	11.4	12.1					7.7	10.8				
2+	65.5	45.9					84.0	76.0				
Enduring impact of conflict: none	26.4	58.7	1.00				25.0	36.4				
1	23.0	11.6	2.77	1.29	5.94	0.009	29.8	36.4	1.08	0.66	1.79	0.754
2+	50.5	29.7	2.77	1.41	5.41	0.003	45.2	27.1	2.19	1.29	3.71	0.004
>1 episode of physical or sexual IPV	80.5	55.7										
Raped by a non-partner	88.5	66.1					14.8	5.0	3.29	1.68	6.44	<0.001
Depressive symptoms	72.4	34.7	3.93	2.14	7.23	<0.001	48.8	28.3	2.17	1.43	3.29	<0.001

** backwards elimination used to improve model fit due to low prevalence of drug use: pstd and traumatic experience in war were tested in model

*** backwards elimination used due to low prevalence of suicidal thoughts: PTSD, traumatic war experiences, experience of non-partner rape ever and of severe IPV were also tested in model

**Table 6:** Prevalence of conflict trauma, perceived enduring impact of conflict, experience of rape and IPV among men and women with (and without) PTSD and depression, and associations between trauma exposure in and after conflict and violence and PTSD and depressive symptoms.

	**Depression in women**						**Depression in men**					
	**Highly symptoms**	**Low symptoms**	**aOR**	**95%CI**		**p value**	**Highly symptoms**	**Low symptoms**	**aOR**	**95%CI**		**p value**
	%	%					%	%				
Traumatic experiences in conflict: none	32.5	45.3	1.00				7.4	14.7	1.00			
1	13.8	11.0	1.23	0.73	2.07	0.446	7.10	11.8	1.13	0.54	2.36	0.743
2+	53.8	43.6	0.97	0.63	1.47	0.873	85.40	73.5	1.80	1.00	3.24	0.052
Enduring impact of conflict: none	43.1	64.0	1.00				28.2	37.3	1.00			
1	13.0	12.3	1.89	1.17	3.03	0.009	31.4	37.3	1.02	0.69	1.50	0.921
2+	43.9	23.8	2.19	1.45	3.31	<0.001	40.4	25.4	1.89	1.24	2.88	0.003
>1 episode of physical or sexual IPV	71.8	48.5	2.06	1.45	2.93	<0.001						
Raped by a non-partner	21.8	12.3	1.61	1.03	2.52	0.035	8.90	5.6	1.29	0.71	2.36	0.404
	**Model of factors associated with depression in women, adjusted for PTSD and alcohol abuse**						**Model of factors associated with depression in men, adjusted for PTSD and alcohol abuse**					
	**High symptoms**	**Low**	**aOR**	**95%CI**		**p value**	**High symptoms**	**Low**	**aOR**	**95%CI**		**p value**
Traumatic experiences in conflict: none	(as above)		1.00				(as above)		1.00			
1			1.20	0.71	2.05	0.499			1.09	0.52	2.29	0.815
2+			0.89	0.58	1.36	0.585			1.34	0.73	2.46	0.338
Enduring impact of conflict: none	(as above)		1.00				(as above)					
1			1.66	1.02	2.68	0.04			0.91	0.61	1.36	0.65
2+			1.69	1.10	2.59	0.016			1.40	0.89	2.18	0.141
Ever >1 episode of physical or sexual IPV	(as above)		1.79	1.25	2.57	0.001						
Ever raped by a non-partner	(as above)		1.31	0.82	2.09	0.26	(as above)		1.26	0.67	2.34	0.474
PTSD symptoms	27.4	5.8	4.50	2.68	7.56	<0.001	38.6	16.7	2.59	1.78	3.77	<0.001
Alcohol abuse	11.8	4.6	2.19	1.14	4.18	0.018	42.7	30.6	1.45	1.04	2.02	0.027

Table 7 shows associations with men’s past year perpetration of violence against women. Exposure to both medium and high level of enduring conflict impact were associated with perpetration of past year rape and physical and/or sexual partner violence, with the effect size increasingly large as the perceived conflict impact exposure was higher. The table also shows that problem drinking, depressive symptoms and past year drug use were all associated with partner violence, and there was some indication that exposure to trauma in conflict may have been too (p = 0.06). Alcohol and drug use were both associated with men’s perpetration of rape in the last year.

**Table 7:** Prevalence of conflict trauma, perceived enduring impact of conflict and mental ill health problems among men who perpetrated phyiscal of sexual IPV in the last year or rape of woman non-partner and those who did not, and associations between trauma exposure in and after conflict and past year perpetration of violence against women.

	Perpetration of physical or sexual partner violence in the last year						Perpetration of rape of a non-partner in the last year					
	IPV (%)	None (%)	aOR	95%CI		p value	Rape (%)	None (%)	aOR	95%CI		p value
Traumatic experiences in conflict: none	4.8	10.7	1.00				7.9	13.9	1.00			
1	7.0	10.9	1.43	0.57	3.58	0.448	7.9	11.2	1.11	0.47	2.62	0.80
2+	88.1	78.5	2.04	0.96	4.36	0.064	84.2	74.9	1.31	0.66	2.60	0.45
Enduring impact of conflict: none	21.6	36.6	1.00				23.2	38.0	1.00			
1	35.8	36.2	1.66	1.06	2.60	0.026	36.9	35.0	1.65	1.04	2.61	0.03
2+	42.5	27.0	2.36	1.43	3.89	0.001	39.8	27.1	2.06	1.23	3.44	0.01
PTSD symptoms	33.0	23.6	1.00	0.65	1.52	0.992	33.3	20.1	1.12	0.73	1.72	0.61
Alcohol abuse	46.0	30.8	1.52	1.06	2.18	0.024	49.6	29.4	2.07	1.43	3.01	<0.001
Depressive symptoms	42.9	27.0	1.51	1.03	2.20	0.033	41.1	28.6	1.22	0.83	1.80	0.31
Past year drug use	25.1	12.5	2.07	1.30	3.28	0.002	31.0	12.7	2.63	1.70	4.06	<0.001

## Discussion

Bougainville, Papua New Guinea has a history of lengthy, violent conflict and our study has shown that not only did it affect and involve a substantial proportion of the current adult population, but it has left an enduring impact. The analysis shows multiple avenues of impact, both on mental health, as well as on perpetration of violence against women and girls. Exposure to the trauma of war is associated with a much greater likelihood of men and women having a high level of PTSD symptoms, and having PTSD is associated with depressive symptoms in men and women and women’s alcohol abuse, and in turn depressive symptoms are associated with suicidal thoughts and drug use as well as alcohol abuse of women and men. We have further shown that a set of experiences which are perceived to indicate an enduring social, emotional and physical (disability) impact of conflict are associated with a higher likelihood of having high levels of PTSD symptoms, depressive symptoms, alcohol abuse, drug use and suicidal thoughts. Perpetration of violence against women is extremely highly prevalent on Bougainville, and we have shown that what is perceived as the enduring social, emotional and physical (disability) impact of conflict is associated with a higher likelihood of rape and partner violence perpetration, as well as the indicators of mental ill-health: high levels of depressive symptoms, drug use and alcohol abuse.

The findings that exposure to human rights abuses and violence during conflict in Bougainville was associated with greater perpetration of violence against women and girls is very much in keeping with the small global literature on this topic[2, 3, 7, 10]. However unusual features of our research are both the lengthy period of time post-conflict over which the impact of war trauma endures, and the finding that beyond exposure to trauma there are perceived to be a range of social, emotional and physical (disability) impacts of conflict which are associated with a greater likelihood of perpetration of violence against women and and of a range of mental health problems which are associated with enduring impact of trauma and are themselves risk factors for violence perpetration. We have not seen a paper with this type of data before.

The impact of service as a military veteran on PTSD and subsequently violence perpetration has been well described, particularly in American veteran populations[8, 9]. The male population studied in Bougainville contained many veterans, but the group was still less than a third (29%) of the total male sample. Whilst combat experiences may have resulted in worse war trauma exposure among combatants, we have shown that prevalence of some form of war trauma exposure was very high across the general population and also especially impacted women, who were very infrequently involved in combat.

The prevalence of mental ill-health was quite high in the Bougainville population, with roughly a third of men and women having high levels of depressive symptomatology. We would hesitate to call this clinical depressed since the short CES-D scale was used but it does indicate a high level of depressive problems. Similarly we would not diagnose clinical PTSD from our assessment of symptoms (especially with the vagueness of the link to specific traumas) but have shown that having many PTSD symptoms was quite common, affecting 15% of women and 25% of men. This prevalence is somewhat higher than that found by Vinck and Pham in Liberia (10.6%) but within the range previously reported from post-conflict countries[2, 33, 34].

The prevalence of gender-based violence found in the study, both past year and during the conflict, were particularly high, but not unexpectedly so. The prevalence was quite similar to that recorded in a survey using comparible measures conducted in the neighbouring (and also post-conflict) Solomon Islands, where 42% of women reported past 12 month physical or sexual partner violence (Secretariat of the Pacific Community 2009). Similarly in Kiribati, 68% of women had ever experience physical or sexual IPV [35]. Sexual violence varies in prevalence across the Pacific with lifetime partner sexual violence ranging from 17% in Tonga to 55% in the Solomons [36]. Very few GBV survivors on Bougainville are able to access health, justice, and social services [37].

Overall our analysis has shown that the impact of conflict on men and women of Bougainville 14 years after the conflict persisted. In the medium term after trauma exposure, the latter’s impact is often interpreted in terms of mental health conditions or disability [38]. This is important in understanding the scope of impact of war and potentially unmet need for medical intervention to improve mental health. In this study we have put a spotlight on the social and emotional impact of war, showing that this persists in Bougainville and is strongly associated with mental ill-health and male perpetration of violence against women. In this way it particularly impacts on women, even though their actual war trauma exposure and combatant experience was much lower than that of men. Whilst some of the experiences perceived as indicative of enduring impact of conflict could have been due to PTSD, we have shown that most of it cannot be explained in those terms as a far lower proportion of women had a potentially clinically significant level of PTSD symptoms than was experiencing enduring conflict impact related to e.g. difficulties with trust or controlling aggression. This study has shown the devastating and lingering impact of conflict on the general population of Bougainville, and the importance of understanding this in understanding the drivers of the islands very high prevalence of violence against women.

### Limitations

The study findings may not have been completely generalizable to Bougainville adults, but the response rate was very high, refusals very few, and the parts of Bougainville that were not included in the sampling frame due to inaccessibility have a very scanty population. It’s unlikely that their conflict experiences would have been much more severe than those who were interviewed. This study has relied on self-reported measures and these may result in under or over-reporting. The very close correlation between past year reports from men and women of violence against women suggests that this is probably reported without much bias. It is often unclear what would be the relationship between survey screening measures of mental illness and clinical diagnosis, but we were not able to validate the measures for Bougainville and make any determination of this. However, they have been well used globally and at least provide a strong indication of the prevalence of symptoms that cause distress and may benefit from treatment. The measures of conflict experience and perceived impacts were developed specifically for the survey and could have missed some aspects of these. However, they did capture the most commonly discussed experiences and impacts. The effect of unique exposure to other experiences in conflict and impact which were not captured would be to bias findings towards the null, but since we have important and significant findings this does not seem to be a major problem undermining the present analysis.

## Conclusions

The war in Bougainville had an incredibly extensive impact on the population. Many people died and its impact on the lives of those who survived it has endured, 14 years after the formal end of hostilities. We have described among the general population, how many live with the memory of traumatic experiences during the war and also perceive a substantial enduring impact post-conflict. This has impacted the mental health of men and women of the islands, and particularly on women’s experience of sexual violence and violence from their male partners. The most powerful way of preventing this destruction would have been if there had not been a conflict.

A major question is: how can the impact of conflict be redressed in Bougainville and violence be prevented. One avenue of work could be through medical mental health interventions, there may be beneficial impact of group cognitive processing therapy, as this has been shown among women conflict sexual trauma survivors[39]. This might positively impact on trust and family or village harmony, but its reach would be severely limited, particularly given the relatively low proportion of the population with very high levels of symptoms. A focus on the social impact may be more important. The social impact of conflict is also read in terms of lost opportunities for education and ultimately income generation, which are particularly visible in the deep poverty of many people on the island. Whilst patriarchal social norms undoubtably have a major role in understanding violence against women, our findings suggest that addressing poverty through meaningful economic opportunities is likely to be an important plank of interventions to prevent violence against women through the impact of wealth and opportunity on emotional well-being.

## Supporting information

S1 File Conflict questions developed for the study. (DOCX)
