# Supplementary material for: Enduring impact of conflict on mental health and gender-based violence perpetration in Bougainville, Papua New Guinea: A cross-sectional study
Source: PLoS One. 2017 Oct 25;12(10):e0186062. doi: 10.1371/journal.pone.0186062 (PMC5656319; doi:10.1371/journal.pone.0186062)
Supplement: S1 File — (DOCX) [file pone.0186062.s001.docx]

**S1 CONFLICT QUESTIONS DEVELOPED FOR THE STUDY**

|  | The following are questions about violence that you may have witnessed or been involved with during the conflict. I know these are very difficult questions and I want to remind you again that everything you say will be confidential. We want to understand what people experienced in the conflict and what impact it has  today on their lives: | |
| --- | --- | --- |
|  | During the conflict years did you: |  |
| 870A | Witness beating? | YES……………………................................…1  NO……………….……….................................2 |
| B | Witness killing? | YES……………………................................…1  NO……………….……….................................2 |
| C | Witness serious injury? | YES……………………................................…1  NO……………….……….................................2 |
| D | Witness rape or sexual violation of women or men? | YES……………………................................…1  NO……………….……….................................2 |
| E | Were you forced into marriage? | YES……………………................................…1  NO……………….……….................................2 |
| F | Were you beaten by the PNGDF or the Resistance or BRA? | YES……………………................................…1  NO……………….……….................................2 |
| G | Were you seriously injured? | YES……………………................................…1  NO……………….……….................................2 |
| H | Were you forced to have sex, raped or otherwise sexual violated? | YES……………………................................…1  NO……………….……….................................2 |
| I | Were you forced to have sex with a family member or friend? | YES……………………................................…1  NO……………….……….................................2 |
| K | Were you detained or imprisoned? | YES……………………................................…1  NO……………….……….................................2 |
| L | Were you beaten or tortured? | YES……………………................................…1  NO……………….……….................................2 |
| M | Were you in a care centre? | YES……………………................................…1  NO……………….……….................................2 |
| N | Were you punished in a care centre because your male relative was in the bush? | YES……………………................................…1  NO……………….……….................................2 |
| O | Were you forced to separate from your parent/spouse? | YES……………………................................…1  NO……………….……….................................2 |
| P | Did you experience verbal or emotional abuse? | YES……………………................................…1  NO……………….……….................................2 |
| Q | Did you experience forced circumcision? | YES……………………................................…1  NO……………….……….................................2 |
| 871A | *(INTENTIONALLY BLANK)* |  |
| B | Did you beat women or participate in beating women? | YES……………………................................…1  NO……………….……….................................2 |
| C | Did you rape, or did you participate in rape of a woman? | YES……………………................................…1  NO……………….……….................................2 |
| D | Did you force, or participate in forcing, a woman into marriage? | YES……………………................................…1  NO……………….……….................................2 |
| E | Did you kill a man or a woman? | YES……………………................................…1  NO……………….……….................................2 |
| F | Were you involved in combat? | YES……………………................................…1  NO……………….……….................................2 |
| 872 | Did you experience any of these things before the conflict? | YES……………………................................…1  NO……………….……….................................2 |
| 873 | Have you experienced any of these things since the conflict? | YES……………………................................…1  NO……………….……….................................2 |
| 874 | Have you witnessed someone experiencing violence after being accused of sorcery? | YES……………………................................…1  NO……………….……….................................2 |
| 875 | Have you participated in violence against someone accused of sorcery? | YES……………………................................…1  NO……………….……….................................2 |
| 876 | Have you experienced violence as a result of being accused of sorcery? | YES……………………................................…1  NO……………….……….................................2 |
|  | Which of the following have you experienced as a result of your exposure to conflict or other trauma: |  |
| 877A | Having to return to school when much older than school age? | YES……………………................................…1  NO……………….……….................................2  NO CHANCE TO RETURN TO SCHOOL……3 |
| B | Unable to complete education? | YES……………………................................…1  NO……………….……….................................2  NO CHANCE TO COMPLETE EDUCATION………………..……3 |
| C | Unable to keep employment? | YES……………………................................…1  NO……………….……….................................2  NO STABLE EMPLOYMENT………….……3 |
| D | Continuing lack of peace in my village or area? | YES……………………................................…1  NO……………….……….................................2 |
| E | Continuing strife in my family? | YES……………………................................…1  NO……………….……….................................2 |
| F | Drinking or using drugs to forget the trauma of conflict? | YES……………………................................…1  NO……………….……….................................2 |
| G | Difficulty having a good relationship with a man? | YES……………………................................…1  NO……………….……….................................2 |
| H | Difficulty controlling my aggression? | YES……………………................................…1  NO……………….……….................................2 |
| I | Difficulty in normal social relations in the community? | YES……………………................................…1  NO……………….……….................................2 |
| J | Unable to trust anyone? | YES……………………................................…1  NO……………….……….................................2 |
| K | Were you disabled as a result of the conflict? | YES……………………................................…1  NO……………….……….................................2 |
| L | Have you gone through a process of redress? | YES……………………................................…1  NO……………….……….................................2 |
| M | Have you gone through a reconciliation process? | YES……………………................................…1  NO……………….……….................................2 |
